# Quantitative Trait Locus Mapping of Resistance to Turnip Yellows Virus in *Brassica rapa* and *Brassica oleracea* and Introgression of These Resistances by Resynthesis Into Allotetraploid Plants for Deployment in *Brassica napus*

**DOI:** 10.3389/fpls.2021.781385

**Published:** 2021-12-10

**Authors:** Shannon F. Greer, Dieter Hackenberg, Vasilis Gegas, Georgia Mitrousia, David Edwards, Jacqueline Batley, Graham R. Teakle, Guy C. Barker, John A. Walsh

**Affiliations:** ^1^School of Life Sciences, University of Warwick, Wellesbourne, United Kingdom; ^2^Limagrain UK Ltd., Rothwell, United Kingdom; ^3^School of Biological Sciences, Institute of Agriculture, The University of Western Australia, Crawley, WA, Australia

**Keywords:** turnip yellows virus, virus resistance, *Brassica rapa*, *Brassica oleracea*, QTL mapping, allotetraploid resynthesis, trait introgression, *Brassica napus*

## Abstract

Turnip yellows virus (TuYV) is aphid-transmitted and causes considerable yield losses in oilseed rape (OSR, *Brassica napus*, genome: AACC) and vegetable brassicas. Insecticide control of the aphid vector is limited due to insecticide resistance and the banning of the most effective active ingredients in the EU. There is only one source of TuYV resistance in current commercial OSR varieties, which has been mapped to a single dominant quantitative trait locus (QTL) on chromosome A04. We report the identification, characterisation, and mapping of TuYV resistance in the diploid progenitor species of OSR, *Brassica rapa* (genome: AA), and *Brassica oleracea* (genome: CC). Phenotyping of F_1_ populations, produced from within-species crosses between resistant and susceptible individuals, revealed the resistances were quantitative and partially dominant. QTL mapping of segregating backcross populations showed that the *B. rapa* resistance was controlled by at least two additive QTLs, one on chromosome A02 and the other on chromosome A06. Together, they explained 40.3% of the phenotypic variation. In *B. oleracea*, a single QTL on chromosome C05 explained 22.1% of the phenotypic variation. The TuYV resistance QTLs detected in this study are different from those in the extant commercial resistant varieties. To exploit these resistances, an allotetraploid (genome: AACC) plant line was resynthesised from the interspecific cross between the TuYV-resistant *B. rapa* and *B. oleracea* lines. Flow cytometry confirmed that plantlets regenerated from the interspecific cross had both A and C genomes and were mixoploid. To stabilise ploidy, a fertile plantlet was self-pollinated to produce seed that had the desired resynthesised, allotetraploid genome AACC. Phenotyping of the resynthesised plants confirmed their resistance to TuYV. Genotyping with resistance-linked markers identified during the mapping in the progenitors confirmed the presence of all TuYV resistance QTLs from *B. rapa* and *B. oleracea*. This is the first report of TuYV resistance mapped in the *Brassica* C genome and of an allotetraploid AACC line possessing dual resistance to TuYV originating from both of its progenitors. The introgression into OSR can now be accelerated, utilising marker-assisted selection, and this may reduce selection pressure for TuYV isolates that are able to overcome existing sources of resistance to TuYV.

## Introduction

*Brassica napus* [oilseed rape (OSR), genome: AACC, 2*n* = 38] is an allotetraploid species that arose from recent (∼7,500 years ago; Chalhoub et al., 2014) and limited interspecific hybridisations between *Brassica rapa* (genome: AA, 2*n* = 20) and *Brassica oleracea* (genome: CC, 2*n* = 18) (Song and Osborn, 1992; Chalhoub et al., 2014). *B. napus* is primarily grown as an oilseed crop but swede, kale, and fodder morphotypes also exist. It is the third most important oil crop after palm (*Elaeis guineensis*) and soybean (*Glycine max*) worldwide (USDA, 2020) and is a major source of vegetable oils, biodiesels, industrial lubricants, and oilseed meals. In 2018, 75 million tonnes of OSR were produced globally, up 31.9% from 56.9 million tonnes in 2008 (FAO, 2018). The largest producers of OSR are Canada, the EU, and China (USDA, 2020). Yet, despite increasing production, winter OSR is currently only reaching a third of its 9.2 t/ha yield potential in the UK (Berry and Spink, 2006). Turnip yellows virus (TuYV) syn. beet western yellows virus (BWYV) is a major contributor to the shortfall in the OSR’s yield potential and has been listed in the top 10 pests and diseases of the crop in a recent survey of 10 countries (Zheng et al., 2020).

Turnip yellows virus is a member of the *Polerovirus* genus in the *Solemoviridae* family. It is transmitted in a persistent, circulative, and non-propagative manner by several aphid species, but the primary and most efficient vector in Europe is the green peach-potato aphid *Myzus persicae* (Schliephake et al., 2000). Up to 72% of winged *M. persicae* caught in yellow water traps at Broom’s Barn Research Centre between 1994 and 2002 were shown to carry TuYV (Stevens et al., 2008), and TuYV incidences in winter OSR between 2007 and 2010 were shown to be closely linked to the numbers of *M. persicae* migrating between August and November when the OSR crops were emerging (Asare-Bediako et al., 2020). TuYV incidences of between 10 and 100% have been reported in OSR grown in the United Kingdom (Walsh et al., 1989; Hardwick et al., 1994; Jay et al., 1999; Asare-Bediako et al., 2020), with higher incidences being reported in recent years due to the ineffectiveness and bans of insecticide treatments that target the aphid vector. Due to the highly polyphagous nature of *M. persicae* and the broad host range of TuYV, both virus and aphid persist in weed species outside of the brassica-growing seasons and during crop rotations (Stevens et al., 1994).

Turnip yellows virus infection can be symptomless or cause a range of non-specific symptoms, such as interveinal yellowing or reddening of the leaves, purpling or reddening of the leaf margins, and stunting that can be mistaken for those caused by abiotic stress. It is, therefore, very difficult to diagnose based on visual symptoms alone. Most notably, TuYV reduces OSR yield, and its impact has been shown to be dependent on infection rates, OSR genotype, and timing of infection (Graichen and Schliephake, 1999; Congdon et al., 2020). In the United Kingdom, TuYV has been reported to reduce OSR yield by as much as 30%, costing the industry upward of £67 million a year, which equates to 9% of the total crop value (Nicholls, 2013). TuYV is not only a problem in the United Kingdom, but it affects other major OSR-producing countries worldwide (Stevens et al., 2008). Yield losses to TuYV of 12–34% have been reported in Germany (Graichen and Schliephake, 1999) and as high as 46% reported in Australia (Jones et al., 2007).

One of the most common ways to control TuYV in brassica crops has been to use insecticides that target the aphid vector (Walsh et al., 1989, 2012; Coutts et al., 2010). However, *M. persicae* has evolved several resistance mechanisms to the most commonly used insecticides (Bass et al., 2014), and the most effective ones, the neonicotinoids, were banned for use on field crops in the EU in 2013 (The European Commission [EC], 2013). It has, therefore, become necessary to explore alternative control methods such as host resistance to the virus.

Currently, there are only two characterised sources of host resistance to TuYV: the resynthesised *B. napus* line “R54” (Graichen, 1994) and the Korean spring *B. napus* variety “Yudal” (Hackenberg et al., 2020). Both resistances were shown to be partial, dominantly inherited, and were mapped to single co-locating QTLs on chromosome A04 (Dreyer et al., 2001; Juergens et al., 2010; Hackenberg et al., 2020). However, further work is needed to determine whether the two resistances are controlled by the same gene. The “R54” resistance has been widely introgressed into commercial OSR varieties, and whilst it may be durable, the wide deployment of this single source increases the selection pressure for resistance-breaking isolates of TuYV.

Due to the recent origin of *B. napus* and extensive breeding programs aimed at improving varieties, the genetic diversity within *B. napus* is limited relative to its diploid progenitor species, *B. rapa* and *B. oleracea*. These diploid species diverged from one another ∼3.8 mya (Inaba and Nishio, 2002). The *B. napus* genetic bottleneck poses a problem when breeding for specific traits such as disease resistance. However, it can be overcome through *B. napus* resynthesis, which involves the interspecific cross of the diploid progenitor species and subsequent genome duplication to produce allotetraploid plants (AACC) that can be used in backcrossing programmes with *B. napus*. *Via* this route, the genetic diversity within *B. rapa* and *B. oleracea* can be combined and exploited in OSR. The resynthesised *B. napus* line “R54” was initially produced as part of a glucosinolate screening program from the interspecific cross of *B. oleracea* “Stone Head” and *B. rapa* “Nr.67” (Gland, 1980). “R54” was later shown to be resistant to TuYV, the resistance having been inadvertently introgressed into the line from its *B. rapa* parent “Nr.67” (Graichen and Peterka, 1995). Resynthesis has also been used to introgress resistance to other pathogens, such as clubroot (*Plasmodiophora brassicae*) and *Verticillium longisporum* from progenitor species into *B. napus* (Diederichsen and Sacristan, 2006; Rygulla et al., 2007).

The aim of this research was to broaden the limited TuYV resistance base in *B. napus* by identifying, characterising, and mapping novel sources of TuYV resistance in the progenitor species of *B. napus* and introgressing them into *B. napus* by resynthesis.

## Materials and Methods

### Plant Lines

In a previously unpublished study, resistance to TuYV was identified in the *B. rapa* ssp. *pekinensis* line ABA15005 and the *B. oleracea* line JWBo12. Mapping populations were developed from within-species crosses using the TuYV-susceptible lines R-o-18 (*B. rapa* ssp. *trilocularis*) and DHSL150 (*B. oleracea*). For QTL analysis of TuYV resistance in line ABA15005, a segregating BC_2_ mapping population SEA17016 was produced, following the crossing strategy in Supplementary Figure 1. For the QTL analysis of the TuYV resistance in *B. oleracea* line JWBo12, a BC_1_ mapping population SEC18031 was produced, following the crossing strategy in Supplementary Figure 2. A TuYV-resistant individual from each of the parental lines, ABA15005 and JWBo12, was self-pollinated to produce S_1_ populations ABA15005a and JWBo12a, respectively.

All plants were cultivated in Levington^®^ M2 compost in an insect-proof, air-conditioned glasshouse at 20±2°C.

### *B. napus* Resynthesis

*Brassica napus* resynthesis was carried out according to Zhang et al. (2004) but with the following modifications. Siliquae produced from the reciprocal interspecific cross between the TuYV-resistant individuals SE4.222 (from the *B. rapa* S_1_ population ABA15005a) and DK1.134 (from the *B. oleracea* line JWBo12), were collected 12–15 days after pollination. They were sterilised in 75% ethanol for 30 s, 5% hypochlorite solution for 15 min, followed by three washes in sterile distilled water. Immature ovules were excised from the sterilised siliquae using a scalpel and forceps on damp filter paper and plated onto Petri dishes of MS (Murashige and Skoog, 1962) regeneration media (1× MS basal salt media and 3% sucrose adjusted to pH 5.8 with 1-M KOH, solidified with 7 g/L phytoagar; after autoclaving, it was supplemented with 1× MS vitamin solution, 400-mg/L glutamine, 2-mg/L 6-benzylaminopurine, and 0.1 mg/L naphthylacetic acid). Petri dishes containing ovules were sealed with micropore tape and left in the dark for 24 h at 20±2°C. They were then transferred to a growth cabinet and ovules cultured at 16 h/8 h (light/dark) and 20±2°C.

Once calli had produced their first true leaves, they were transplanted into deep-well Phytatrays™ (Merck Life Science UK Ltd., Dorset, United Kingdom), containing MS rooting media (^1^/_2_× MS basal salt media and 2% sucrose adjusted to pH 5.8 with 1-M KOH, solidified with 7 g/L phytoagar; after autoclaving, it was supplemented with ^1^/_2_× MS vitamin solution and 1-mg/L Indole-3-butyric acid). The base of the Phytatrays™ was wrapped in foil to mimic the darkness of soil and returned to the growth cabinet. Plantlets were removed from the Phytatrays™ once abundant roots had developed and excess media was washed from their roots using distilled water. They were then repotted into Levington^®^ M2 compost and covered with clear plastic bags secured to the pots using an elastic band. Sequential corners of the plastic bags were cut off at intervals of 1/2 days to facilitate acclimatisation to atmospheric humidity.

Regenerated plantlets (RP1, RP2, and RP3) were propagated by taking cuttings. Five cuttings were taken from RP1 (C1–C5) and RP2 (C6–C10), and two cuttings were taken from RP3 (C11 and C12). Secondary shoots from plants were cut from the primary shoot close to their base using a scalpel. The base of the cuttings was then dipped in Strike2 rooting hormone (Bayer Garden, Monheim am Rhein, Germany), planted in separate wells of 40-well trays (4 cm × 4 cm × 5 cm) filled with Levington^®^ F2S compost and covered with a clear Perspex^®^ lid to retain humidity. Cuttings were grown in the glasshouse at 16 h/8 h (light/dark) at 20±2°C. When the cuttings had well-developed roots, they were transplanted into FP7 (7 cm × 7 cm × 8 cm) pots filled with Levington M2^®^ compost and left to establish in the glasshouse for at least four weeks. The roots of the cuttings were then treated with 0.34% colchicine for 90 min according to Fletcher et al. (1998) to stimulate chromosome multiplication. The original regenerated plantlets, from which the cuttings were taken, were not treated with colchicine. An S_1_ population SER19001 was produced by self-pollinating an individual colchicine-treated cutting.

### Ploidy Testing

The ploidy of regenerated plantlets, colchicine-treated cuttings, individuals belonging to the resynthesised allotetraploid AACC S_1_ population SER19001 and *Brassica* control lines was determined on two to four fresh leaves from each plant by Plant Cytometry Services (PCS, Didam, Netherlands) using flow cytometry. Ploidy ratios for each plant were determined against PCS’s standards, *Vinca minor* or *Chlorophytum comosum*, and compared to a range of *Brassica* control lines. The *Brassica* control lines included the parental lines of the interspecific cross (ABA15005a and JWBo12) and additional *B. rapa* (R-o-18), *B. oleracea* (A12DHd), and *B. napus* (Anastasia) lines, which were of known ploidy.

### Turnip Yellows Virus Resistance Phenotyping

The TuYV isolate W2016FE was used for resistance phenotyping and originated from cabbage grown in a field trial at Wellesbourne, United Kingdom in 2016. This isolate belongs to the largest and most common phylogenetic group of TuYV (Newbert, 2016) based on unpublished sequence analysis of its P0 gene. The isolate was maintained in OSR variety Castille in an insectary at 16 h/8 h (light/dark) and 18±2°C by serial transmission using *M. persicae* clone Mpn1 (Blackman et al., 2007). Phenotyping for resistance and susceptibility to TuYV involved exposing plants to viruliferous *M. persicae* (10 aphids/plant), as described by Hackenberg et al. (2020). The aphids were left on the plants for 10 days and were then killed using foliar insecticide sprays of 0.4 ml/L lambda-cyhalothrin (Hallmark Zeon, Syngenta, Fulbourn, United Kingdom) and 0.75 g/L pyridine azomethine (Plenum, Syngenta, Fulbourn, United Kingdom) and a systemic drench of 0.166 g/L, thiamethoxam (Actara, Syngenta, Fulbourn, United Kingdom). The aphid transmission experiments were carried out in a glasshouse compartment at 18±2°C.

Turnip yellows virus titre was quantified in plants using triple antibody sandwich enzyme-linked immunosorbent assay (TAS-ELISA) (Hunter et al., 2002). All wash steps for the TAS-ELISA were carried out using the Wellwash™ 5000 plate washer (Denley) and Phosphate Buffered Saline – Tween^®^20 (PBS-T, 130-mM NaCl, 1.3-mM KCL, 0.5-mM KH_2_PO_4_, 3.2-mM Na_2_HPO_4_, and 0.05% Tween^®^20). The primary antibody (IgG-AS0049, DSMZ) was diluted 1:1,000 in a carbonate buffer (15-mM Na_2_CO_3_ and 350-mM NaHCO_3_) and 200 μl incubated in each well of a 96-well plate (Nunc MaxiSorp™, Sigma-Aldrich^®^) for 4 h at 36°C. During the primary antibody incubation, the youngest leaf from each plant was sampled and mechanically macerated between two metal rollers (Leaf Juice Press, Meku-Pollaehne) and sap collected. The plates were washed three times to remove the primary antibody, and 150 μl sap was pipetted into duplicate wells for each sample. Sap from a plant known to be infected with TuYV (the inoculum source) was used as a positive control and was pipetted into duplicate wells. Sap from a healthy plant was used as a negative control standard (NCS) and was pipetted into six wells of each plate. The plates were incubated overnight at 4°C. Plates were washed three times to remove the sap. The secondary antibody (mouse Mab-AS0049/1, DSMZ) was diluted 1:1,000 in PBS-T-BSA (PBS-T with 2.5-g/L Bovine Serum Albumin, Sigma-Aldrich^®^) and 150 μl pipetted into each well and incubated at 36°C for 2 h. Both the primary and secondary antibodies are specific to the major coat protein of TuYV. The plates were washed, and 150 μl of the tertiary antibody (IgG anti-mouse-A3526, Sigma-Aldrich^®^) diluted 1:5,000 in PBST-BSA was pipetted into each well and incubated for 2 h at 36°C. The tertiary antibody is specific to the secondary antibody and is conjugated to alkaline phosphatase. The plates were washed, and 150 μl of substrate solution [(1 mg/1 ml 4-Nitrophenyl phosphate disodium salt hexahydrate, Sigma-Aldrich®), 9% diethanolamine (Sigma-Aldrich®), titrated to pH 9.8 with HCl)] was added to each well and incubated at room temperature. Absorbances (A_405_ values) were read on a Biochem Anthos 2010 plate reader at 405 nm with a reference filter of 620 nm. Plate readings were exported to Microsoft Excel for analysis. Duplicate readings were averaged to obtain a single A_405_ value per sample. To standardise readings between plates, the absorbance values of experimental samples on a specific plate (*x*) were multiplied by the factor (*y*), calculated using the formula:


$$y=\frac{M\,e\,a\,n\,o\,f\,N\,C\,S\,o\,n\,a\,l\,l\,p\,l\,a\,t\,e\,s}{M\,e\,a\,n\,o\,f\,N\,C\,S\,o\,n\,p\,l\,a\,t\,e\,x}$$

For TuYV resistance QTL mapping, 203 individuals from the *B. rapa* BC_2_ population SEA17016 and 200 individuals from the *B. oleracea* BC_1_ population SEC18031 were challenged with viruliferous aphids, alongside 5–20 individuals (dependent on seed availability) from the TuYV-resistant and -susceptible parental lines. TuYV titre in plants was quantified in SEA17016 at 3 weeks and in SEC18031 at 6 weeks post-challenge. It was not possible to quantify TuYV titre in the *B. oleracea* and *B. rapa*-mapping populations at similar time points because the *B. rapa* populations were rapid cycling. They began to bolt shortly after the ELISA was carried out at 3 weeks post-challenge, but, at this time point, the viral titre had not yet accumulated to detectable levels in the *B. oleracea* populations, and thus, the ELISA was carried out later for the *B. oleracea* populations. ELISA was not carried out on bolting plants to avoid the effects of age-onset resistance.

The TuYV resistance status of the resynthesised allotetraploid AACC S_1_ population SER19001 was determined by phenotyping 12 individuals alongside 18 individuals from a range of control lines. The control lines included an S_1_ population from each of the TuYV-resistant *B. rapa* (ABA15005a) and *B. oleracea* (JWBo12a) parental lines, TuYV-susceptible *B. rapa* (R-o-18), and *B. oleracea* (DHSL150) lines, and *B. rapa* (ABA15010) and *B. oleracea* (SEC17008) F_1_ populations derived from the crosses between the TuYV-resistant and –susceptible lines. Two individuals from each line were not challenged with TuYV, as healthy controls. Due to the limited availability of seed, only eight ABA15005a individuals were phenotyped. TuYV titre was quantified in all lines at four weeks post TuYV challenge using TAS-ELISA; the TuYV titre in SER19001 and the *B. oleracea* lines was also quantified at 11 weeks post-challenge. The *B. rapa* lines did not have sufficient leaf material and were flowering at 11 weeks post-challenge, so it was not possible to test them. For ELISAs at both time points, A_405_ values were normalised to the healthy control before statistical comparisons were made. This was done by subtracting the average A_405_ value of the healthy control plants of a given line from A_405_ values of individuals from the same line. Statistical comparisons and normality testing of TuYV titres (A_405_ values) for the different plant lines were carried out in IBM^®^ SPSS^®^ Statistics version 25.

### Genotyping and Quantitative Trait Locus Analysis

Genomic DNA (gDNA) was isolated from 80 individuals from the *B. rapa* BC_2_ population SEA17016, 115 individuals from the *B. oleracea* BC_1_ population SEC180131, and the parental lines of these populations, by the LGC Group (Hoddesdon, United Kingdom). Individuals were then subsequently genotyped using the *Brassica* 60K Bead Chip Array (Infinium, Illumina Inc., San Diego, CA, United States) (Clarke et al., 2016) at The University of Western Australia. For both populations, a genetic linkage map was constructed, and QTL(s) associated with TuYV resistance were identified, using the R/qtl package in RStudio 1.0.153 (Broman and Sen, 2009; Broman, 2010). Only polymorphic DNA markers that had less than 20% missing calls and that did not deviate significantly (*p* < 0.05) from the expected segregation ratio (1:1 for a backcross population) were used to construct the genetic linkage maps. Missing data were inspected using the “plotMissing” and “nmissing” functions in R/qtl. Segregation patterns for each marker were inspected using the “geno.table.” Markers with duplicate genotypes were identified using the “findDupMarkers” function and removed to create the minimal maps. Markers were assigned to the same linkage group if they had an associated LOD score >6 and a recombination fraction <0.35. Genetic distances in centimorgans (cM) between the markers for each linkage group were calculated using the “orderMarkers” function (Haldane, 2008). Marker order was optimised to minimise obligate crossover events using the “ripple” function, a sliding window of four markers, and a genotyping error probability of 0.005. The distribution of A_405_ values in SEA17016 and SEC18031 populations deviated significantly from normality, so were transformed using log_10_. One-dimensional interval mapping (IM) (“scanone” function) was performed on untransformed and transformed A_405_ values, using a non-parametric Kruskal–Wallis algorithm (Kruglyak and Lander, 1995) and parametric Haley–Knott regression (Haley and Knott, 1992), respectively. Two-dimensional IM (“scantwo” function) was performed for both populations using transformed A_405_ values and Haley–Knott regression. For both one- and two-dimensional IM, genome-wide LOD significance (LOD_*GWS*,_ α < 0.05) was determined by permutation test with 1,000 permutations. QTL intervals (“lodint” function) of 1.5 LOD were calculated from the peak LOD score and extended to the next adjacent marker.

### Genomic DNA Extraction and PCR Genotyping

gDNA was extracted from leaf tissue following Dellaporta et al. (1983). gDNA was extracted from the resynthesised allotetraploid AACC S_1_ population SER19001, the TuYV-resistant parental individuals SE4.222 (from *B. rapa* S_1_ population ABA15005a) and DK1.134 (from *B. oleracea* line JWBo12), and TuYV-susceptible *B. rapa* (R-o-18) and *B. oleracea* (DHSL150) control lines. TuYV resistance-linked markers identified from mapping in the diploid species were then amplified from these individuals by PCR. PCR amplification was carried out in 25 μl reactions using 50 ng gDNA, high-fidelity Phusion™ polymerase (Fisher Scientific UK Ltd., Loughborough, United Kingdom) according to the manufacturer’s instructions and primers specific to each marker. Primers for the A genome markers, Bn-A02-p7840077 (forward: 5′-CGTATTCTATGATTAAGG-3′, reverse: 5′-TCAAAGGTAACAATCTAG-3′, T_*m*_ = 50°C) and Bn-A06-p18369013 (forward: 5′-AACAAACAGAAAGCTTGG-3′, reverse 5′-TCAAGTGACGGTTTATGG-3′, T_*m*_ = 56°C) were designed using the *B. rapa* Chiifu-401-42 reference genome (NCBI: GCA_000309985.3). Primers for the C genome marker, Bn-scaff_16082_1-p278297 (forward: 5′-TCTTCGGTCAGTAGAACG-3′, reverse: 5′-GCGAAATATACAATGTGG-3′, T_*m*_ = 54°C) were designed using the *B. oleracea* TO1000 reference genome (NCBI: GCA_000695525.1). The reaction mixtures were amplified at 98°C for 30 s followed by 35 cycles of 98°C for 15 s, a T_*m*_ specific to the primer pair (50–56°C) for 30 s and 72°C for 30 s. The amplifications were finished with a hold at 72°C for 10 min. Amplicons were then Sanger sequenced.

## Results

### Quantitative Trait Locus Mapping of Turnip Yellows Virus Resistance in *B. rapa* Line ABA15005

**FIGURE 1:**
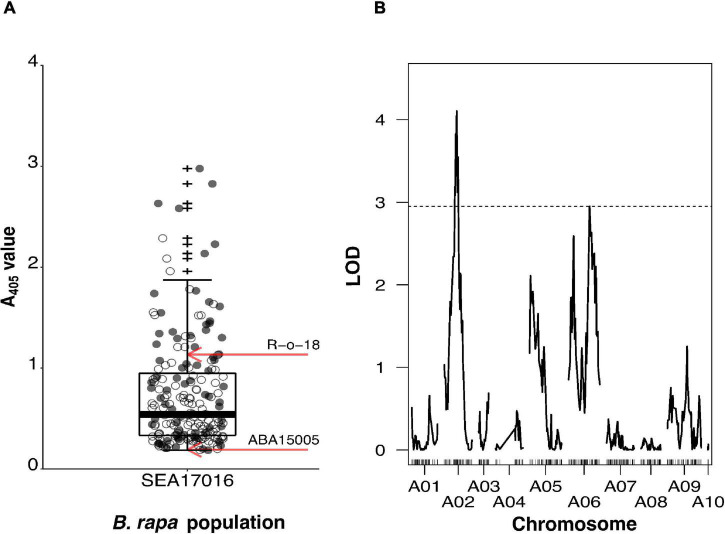
Mapping of turnip yellows virus (TuYV) resistance from *Brassica rapa* line ABA15005 using the segregating BC_2_ population SEA17016. SEA17016 was produced from the cross R-o-18 × BC_1_, where the BC_1_ plant was resistant to TuYV. **(A)** Enzyme-Linked Immunosorbent Assay A_405_ values for BC_2_ population SEA17016 (*n* = 203) and mean values for S_1_ populations of the TuYV-resistant parental line ABA15005 (*n* = 5) and TuYV-susceptible parental line R-o-18 (*n* = 12) indicated by red arrows. Individuals subsequently genotyped for quantitative trait locus (QTL) analysis are indicated as grey points. **(B)** The LOD plot of QTL analysis of BC_2_ population SEA17016, using normalised log_10_ (A_405_) values and Haley–Knott one-dimensional interval mapping. Genome-wide significance LOD threshold (α≤0.05) of 1,000 permutations is indicated by a dashed horizontal line. Outliers are indicated by +.

The BC_2_ population SEA17016 segregated for TuYV resistance. Individuals displayed a continuous, broad range of A_405_ values (0.185–2.977), and 83% of them had an A_405_ value intermediate to the mean A_405_ values of the S_1_ population (ABA15005a) of the resistant parent and the susceptible parent (R-o-18) (Figure 1A). A Shapiro–Wilk test showed that ELISA A_405_ values for ABA15005a (*p* = 0.805) and R-o-18 (*p* = 0.119) were normally distributed. A_405_ values for the 203 BC_2_ individuals of the mapping population (SEA17016) were not normally distributed (*p* < 0.001), with a skewness of 1.690 (SE = 0.171) and kurtosis of 3.139 (SE = 0.340). Welch’s *t*-test showed that ABA15005a (mean A_405_ ± SD = 0.191 ± 0.029, *n* = 5) had a significantly lower mean A_405_ value than R-o-18 (mean A_405_ ± SD = 1.212 ± 0.599, *n* = 12), *t*(11.125) = −5.886, *p* < 0.001.

A subset of 72 individuals from the BC_2_ population, representing the spread of A_405_ values in the population (Figure 1A), was genotyped alongside the parents for QTL analysis. Of the 77,970 markers on the *Brassica* 60K genotyping array, 21,219 had less than 20% missing calls. Of these, 2,053 had a missing genotype call for one or both of the parents and so were discarded. Of the remaining markers, 14,108 were monomorphic between the parents, and 1,960 markers were heterozygous for one or both of the parents so were also discarded. A further 619 had a segregation ratio that deviated significantly from the expected 1:1 for a backcross population. Removal of these markers resulted in 2,479 useful markers; of which, 351 represented the overall recombination within SEA17016 and were used to make the minimal genetic map. This had a total length of 868.1 cM and comprised of 10 linkage groups, which represented the 10 *B. rapa* chromosomes (A01–A10) (Supplementary Figure 3A). Markers in the minimal map only segregated where the BC_1_ parent of the BC_2_ population was heterozygous (Supplementary Figure 3B). This meant that portions of linkage groups, most noticeably of A03, A04, and A10, were not represented in the minimal map as the BC_1_ parent was homozygous for the TuYV-susceptible R-o-18 genotype at these locations. The average distance between markers in the map was 2.5 cM, with the minimum inter-marker distance being 0.6 cM and the maximum being 56.2 cM. The largest inter-marker distance was on A04 and was a result of markers located in the centre of A04, not segregating in the BC_2_ population. The smallest linkage group at 1.8 cM was A10 and comprised of three markers. This was because the BC_1_ parent was homozygous for the R-o-18 genotype for almost the entirety of A10. The largest linkage group in terms of length was A09, which was 124.3 cM (46 markers), and in terms of marker number, was A07 (53 markers, 101.9 cM).

Non-parametric (Kruskal–Wallis) one-dimensional interval mapping (IM) was carried out on untransformed A_405_ values, and parametric (Haley–Knott) one-dimensional IM was carried out on transformed log_10_ (A_405_) values (Shapiro–Wilk test *p* = 0.053), using the minimal map of the BC_2_. The outputs from both analyses were not found to be significantly different from one another (Supplementary Figure 4); hence, log_10_ (A_405_) values were used for subsequent two-dimensional IM and QTL modelling, for which only parametric methods are available. One-dimensional IM using log_10_ (A_405_) values identified two significant QTLs on chromosome A02 at 45.9 cM (LOD = 4.10) and A06 at 78.0 cM (LOD = 2.96) that surpassed the genome-wide significance threshold (LOD_*GWS*_) of 2.95 (Figure 1B). Markers Bn-A02-p7840077 and Bn-A06-p18369013 positioned at or closest to the peak LOD score positions on A02 and A06, respectively. The QTL identified on A02 explained 23.1% of the phenotypic variation, the QTL on A06 explained 17.2%, and together, they explained 40.3% (Table 1). The QTL on A02 had a 1.5-LOD interval of 16.0 cM that spanned from 37.0 to 53.0 cM and was flanked by the markers Bn-A02-p6627382 and Bn-A02-p13123802. The QTL on A06 had a 1.5-LOD interval of 34.5 cM that spanned from 69.0 to 103.5 cM and was flanked by the markers Bn-A06-p18144127 and Bn-A06-p24156940.

**TABLE 1:** Details of quantitative trait loci (QTL) for turnip yellows virus (TuYV) resistance in *Brassica rapa* BC_2_ population SEA17016 and *Brassica oleracea* BC_1_ population SEC18031.

Population	LOD_GWS_	Peak LOD	Position of peak LOD	1.5 LOD QTL interval	PVE (%)	Additive effect^a^	Flanking markers^b^	*n*
SEA17016	2.95	4.10	A02 at 45.9 cM	37.0 cM–53.0 cM	23.1	−0.507	Bn-A02-p6627382 and Bn-A02-p13123802	72
		2.96	A06 at 78.0 cM	69.0 cM–103.5 cM	17.2	−0.485	Bn-A06-p18144127 and Bn-A06-p24156940	
SEC18031	2.77	3.06	C05 at 47.3 cM	41.0 cM–88.0 cM	11.5	−0.323	Bn-scaff_18181_1-p620712 and	115
		2.81	C05 at 81.4 cM		10.6	−0.321	Bn-scaff_23186_1-p18537	

*^a^Based on untransformed A_405_ means for each genotypic group.*

*^b^The marker sequences are available at CropSNPdb – http://snpdb.appliedbioinformatics.com.au/.*

Subsequent two-dimensional IM supported the two QTL model involving chromosomes A02 and A06, predicted by the one-dimensional IM. A significant full model, with two positively interacting QTLs was identified, involving the QTLs detected by one-dimensional IM on chromosomes A02 at 45.9 cM and A06 at 78.0 cM (LOD_full_ = 7.25, LOD_full_ _GWS_ = 6.00, *p* = 0.009). Another significant additive model, with two QTLs that did not interact was also identified by this method, again involving the original QTL on chromosome A02 at 45.9 cM and a different QTL on chromosome A06, this time, at 18.6 cM (LOD_add_ = 7.02, LOD_add_ _GWS_ = 5.03, *p* = 0.014). A LOD peak was observed on chromosome A06 at 18.6 cM (LOD = 2.59) during one-dimensional IM, but it did not reach the LOD_GWS_ (2.95). No other significant two-QTL models were detected.

### Quantitative Trait Locus Mapping of Turnip Yellows Virus Resistance in *B. oleracea* Line JWBo12

**FIGURE 2:**
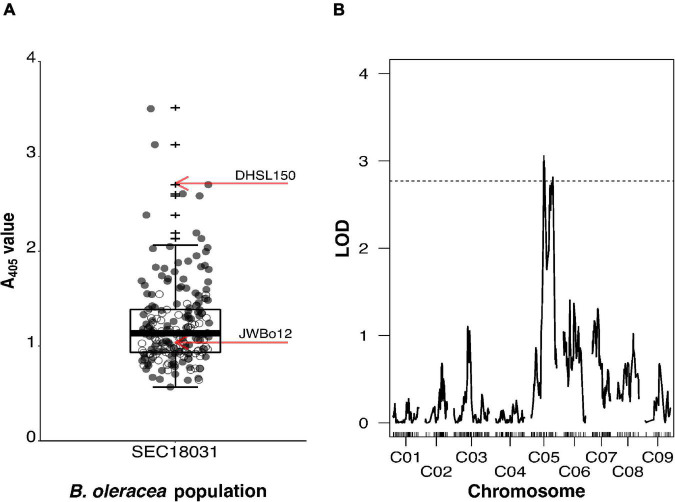
Mapping of TuYV resistance from *Brassica oleracea* line JWBo12 using the segregating BC_1_ population SEC18031. SEC18031 was produced from the cross DHSL150 × F_1_, where the F_1_ plant was resistant to TuYV. **(A)** Enzyme-Linked Immunosorbent Assay A_405_ values for BC_1_ population SEC18031 (*n* = 200) and mean values for S_1_ populations of the TuYV-resistant parental line JWBo12 (*n* = 7) and TuYV-susceptible parental line DHSL150 (*n* = 18) indicated by red arrows. Individuals subsequently genotyped for quantitative trait locus (QTL) analysis are indicated as grey points. **(B)** The LOD plot of QTL analysis of BC_1_ population SEC18031, using normalised log_10_ (A_405_) values and Haley–Knott one-dimensional mapping. Genome-wide significance LOD threshold (α≤0.05) of 1,000 permutations is indicated by a dashed horizontal line. Outliers are indicated by +.

The BC_1_ population SEC18031 segregated for TuYV resistance. Individuals displayed a continuous, broad range of A_405_ values (0.566–3.500), and 60% of them had an A_405_ value intermediate to the mean A_405_ values of the S_1_ population (JWBo12a) of the resistant parent and the susceptible parent (DHSL150) (Figure 2A). A Shapiro–Wilk test showed that ELISA A_405_ values for JWBo12a (*p* = 0.495) and DHSL150 (*p* = 0.123) were normally distributed. A_405_ values for the 200 BC_1_ individuals of the mapping population (SEC18031) were not normally distributed (*p* < 0.001), with a skewness of 1.871 (SE = 0.172) and kurtosis of 5.361 (SE = 0.342). Welch’s *t*-test showed that JWBo12a (mean A_405_ ± SD = 1.037 ± 0.331, *n* = 7) had a significantly lower mean A_405_ value than DHSL150 (mean A_405_ ± SD = 2.714 ± 0.642, *n* = 18), *t*(20.752) = −8.551, *p* < 0.001.

A subset of 115 individuals from the BC_1_ population, representing the spread of A_405_ values in the population (Figure 2A), was genotyped alongside the parental lines for QTL analysis. Of the 77,970 markers on the *Brassica* 60K genotyping array, 27,422 had less than 20% missing calls. Of these, 1,286 had a missing genotype call for one or both of the parents and so were discarded. Of the remaining markers, 18,869 were monomorphic between the parents, and 2,191 markers were heterozygous for one or both of the parents so were also discarded. A further 667 markers had a segregation ratio that deviated significantly from the expected 1:1 for a backcross population. Removal of these markers resulted in 4,409 useful markers, of these, 448 represented the overall recombination within SEC18031 and were used to make the minimal genetic map. This had a total length of 837.1 cM and comprised of nine linkage groups, which represented the nine *B. oleracea* chromosomes (C01–C09) (Supplementary Figure 5). The average distance between markers in the map was 1.9 cM, with the minimum inter-marker distance being 0.4 cM and the maximum being 27.0 cM. The smallest linkage group in terms of length was C07, which was 68.4-cM long (49 markers), and the smallest in terms of marker number was C09 (93.2 cM, 26 markers). The largest linkage group C03 was 131.3 cM in length and comprised the most markers (84).

As with the *B. rapa* mapping, non-parametric (Kruskal–Wallis) one-dimensional IM was carried out on untransformed A_405_ values, and parametric (Haley–Knott) one-dimensional IM was carried out on transformed log_10_ (A_405_) values (Shapiro–Wilk test *p* = 0.764), using the minimal map for SEC18031. The outputs from both analyses were not found to be significantly different from one another (Supplementary Figure 6), hence log_10_ (A_405_) values were used for subsequent two-dimensional IM and QTL modelling. The two-dimensional IM did not identify any significant two-QTL models. The one-dimensional IM using the log_10_ (A_405_) values identified two distinct and significant QTLs on chromosome C05 at 47.3 cM (LOD = 3.06) and 81.4 cM (LOD = 2.81) that surpassed the LOD_*GWS*_ of 2.77 (Figure 2B). Markers Bn-scaff_16082_1-p278297 and Bn-scaff_20219_1-p220228 positioned at the peak LOD score positions on C05 at 43.7 cM and 81.4 cM, respectively. The QTL identified at 47.3 cM explained 11.5% of the phenotypic variation, the QTL at 81.4 cM explained 10.6%, and together, they explained 22.1% (Table 1). The 1.5-LOD intervals of each QTL overlapped. They spanned from 41.0 to 88.0 cM on C05, a total interval length of 47.0 cM and were flanked by the markers Bn-scaff_18181_1-p620712 and Bn-scaff_23186_1-p18537.

### Development of Allotetraploid AACC With Novel Turnip Yellows Virus Resistances From *B. rapa* and *B. oleracea*

#### *B. napus* Resynthesis

A reciprocal, interspecific cross was carried out using TuYV-resistant individuals from *B. rapa* S_1_ population ABA15005a and *B. oleracea* line JWBo12. ABA15005a was produced by self-pollinating a TuYV-resistant individual from ABA15005. The interspecific cross was more productive when ABA15005a was used as the female parent (7.4 ovules/siliqua) compared to when it was used as the male parent (0.7 ovules/siliqua). Of the 243 ovules collected for the cross ABA15005a × JWBo12, 2.1% germinated (five ovules), and 1.2% (three ovules) were regenerated into rooted plantlets (RP1, RP2, and RP3). In comparison, fewer ovules were rescued from the reciprocal cross JWBo12 × ABA15005a (17 ovules); despite the fact a greater proportion (58.8%) germinated, none regenerated into rooted plantlets.

ABA15005a had an average ploidy ratio (PR) (PR ± SD = 0.609 ± 0.006, *n* = 4) similar to that of the *B. rapa* standard R-o-18 (PR ± SD = 0.606 ± 0.008, *n* = 4) and JWBo12 had an average ploidy ratio (PR ± SD = 0.813 ± 0.005, *n* = 4) similar to that of the *B. oleracea* standard A12DHd (PR ± SD = 0.818 ± 0.006, *n* = 4) (Table 2), confirming they had the diploid *Brassica* AA and CC genomes, respectively. The regenerated plantlets RP1 and RP2 were mixoploid; the diploid cells had average ploidy ratios (PR ± SD = 0.712 ± 0.004, *n* = 2 and PR ± SD = 0.704 ± 0.007, *n* = 2, respectively) intermediate to the parental lines, indicating they had the allodiploid *Brassica* genome AC (Table 2). The ploidy of RP3 could not be determined as it had died before testing. Ploidy ratios of allotetraploid AACC (PR = ∼1.4) and 4 × AC (PR = ∼2.8) were detected in RP1 and RP2 alongside the AC allodiploid (PR = ∼0.7) (Table 2), indicating that spontaneous chromosome multiplication had occurred in these plantlets. Following colchicine treatment, all of the cuttings taken from RP1-3 were shown to be mixoploid with ploidies of AC to 16 × AC detected, indicating that the treatment had enhanced chromosome multiplication (Table 2). For example, ploidy ratios of up to 16 × AC (PR = ∼11.2) were detected in colchicine-treated cuttings C9 and C10 (from RP2), but the maximum ploidy ratio detected in the untreated regenerated plantlets was 4 × AC (Table 2). Different ploidies were detected within and between leaf samples taken from the same regenerated plantlets or cuttings.

**TABLE 2:** Ploidy of colchicine-treated cuttings (C1–12) taken from regenerated plantlets (RP1–3), produced from the interspecific cross of TuYV-resistant lines ABA15005a (*B. rapa*) and JWBo12 (*B. oleracea*).

Plant line/individual	Cutting	No. leaves tested	Ploidy (inferred from ratio to the standards)	Average ± SD*^a^* ploidy ratio to *Vinca minor*	Percentage ± SD of nuclei with ploidy				
					1 × AC	2 × AC	4 × AC	8 × AC	16 × AC
R-o-18 (standard)	–	4	AA	0.606 ± 0.008	–	–	–	–	–
A12DHd (standard)	–	4	CC	0.818 ± 0.006	–	–	–	–	–
Anastasia (standard)	–	4	AACC	1.375 ± 0.006	–	–	–	–	–
ABA15005a	–	4	AA	0.609 ± 0.006	–	–	–	–	–
JWBo12	–	4	CC	0.813 ± 0.005	–	–	–	–	–
RP1	–	2	AC mixoploid	0.712 ± 0.004	39.5 ± 8.4	52.3 ± 8.6	8.2 ± 0.2	0.0 ± 0.0	0.0 ± 0.0
	C1	4	AC mixoploid	–	29.6 ± 28.3	58.4 ± 20.7	12.0 ± 8.9	0.0 ± 0.0	0.0 ± 0.0
	C2	4	AC mixoploid	–	56.4 ± 11.9	37.7 ± 10.6	5.9 ± 2.3	0.0 ± 0.0	0.0 ± 0.0
	C3	4	AC mixoploid	–	17.8 ± 21.3	60.1 ± 7.4	22.1 ± 16.0	0.0 ± 0.0	0.0 ± 0.0
	C4	4	AC mixoploid	–	34.0 ± 20.9	51.2 ± 15.4	14.8 ± 12.8	0.0 ± 0.0	0.0 ± 0.0
	C5	3	AC mixoploid	–	10.2 ± 17.7	67.4 ± 13.2	22.4 ± 6.5	0.0 ± 0.0	0.0 ± 0.0
RP2	–	2	AC mixoploid	0.704 ± 0.007	75.6 ± 17.2	22.3 ± 16.6	2.1 ± 0.7	0.0 ± 0.0	0.0 ± 0.0
	C6	4	AC mixoploid	–	31.5 ± 20.0	53.1 ± 11.2	15.4 ± 9.9	0.0 ± 0.0	0.0 ± 0.0
	C7	4	AC mixoploid	–	51.2 ± 19.7	44.5 ± 17.6	4.3 ± 2.3	0.0 ± 0.0	0.0 ± 0.0
	C8	4	AC mixoploid	–	50.4 ± 23.3	43.7 ± 20.2	5.9 ± 4.2	0.0 ± 0.0	0.0 ± 0.0
	C9	4	AC mixoploid	–	10.4 ± 20.8	18.9 ± 23.0	36.4 ± 19.9	27.7 ± 22.8	6.6 ± 9.2
	C10	4	AC mixoploid	–	20.1 ± 24.3	32.1 ± 23.2	29.9 ± 25.7	16.6 ± 17.7	1.3 ± 1.5
RP3	–	0	Unknown	–	–	–	–	–	–
	C11	4	AC mixoploid	–	49.1 ± 14.6	45.4 ± 13.5	5.2 ± 1.6	0.3 ± 0.5	0.0 ± 0.0
	C12	4	AC mixoploid	–	25.5 ± 19.6	41.6 ± 6.2	27.2 ± 10.0	5.7 ± 8.6	0.0 ± 0.0

*^a^SD, standard deviation.*

To stabilise ploidy, identify allotetraploids, and obtain seed, cuttings were self-pollinated. All flowered after eight weeks of vernalisation, but only one cutting C10 (from RP2) produced fertile flowers. It was self-pollinated to produce the S_1_ population SER19001 (*n* = 7). When ploidy was tested, all seven individuals from SER19001 had a ploidy ratio similar to that of the *B. napus* OSR standard, Anastasia (PR ± SD = 0.138 ± 0.001), indicating they had the desired allotetraploid genome AACC (Table 3).

**TABLE 3:** Ploidy of SER19.1–7 S_1_ individuals from population SER19001 derived from the interspecific cross of TuYV-resistant lines ABA15005a (*B. rapa*) and JWBo12 (*B. oleracea*).

Plant line or individual	*n* ^a^	Ploidy (inferred from ratio to the standards)	Average ± SD^b^ ploidy ratio to *Chlorophytum comosum*
R-o-18 (standard)	2	AA	0.062 ± 0.000
A12DHd (standard)	2	CC	0.082 ± 0.000
Anastasia (standard)	2	AACC	0.138 ± 0.001
ABA15005a	2	AA	0.060 ± 0.001
JWBo12	2	CC	0.084 ± 0.000
SER19.1	2	AACC	0.137 ± 0.002
SER19.2	2	AACC	0.137 ± 0.000
SER19.3	2	AACC	0.137 ± 0.000
SER19.4	2	AACC	0.140 ± 0.001
SER19.5	2	AACC	0.130 ± 0.001
SER19.6	2	AACC	0.135 ± 0.001
SER19.7	2	AACC	0.136 ± 0.002

*^a^n – number of leaf samples tested per plant.*

*^b^SD, standard deviation.*

#### Phenotyping Resynthesised Allotetraploid AACC Plants for Turnip Yellows Virus Resistance

**FIGURE 3:**
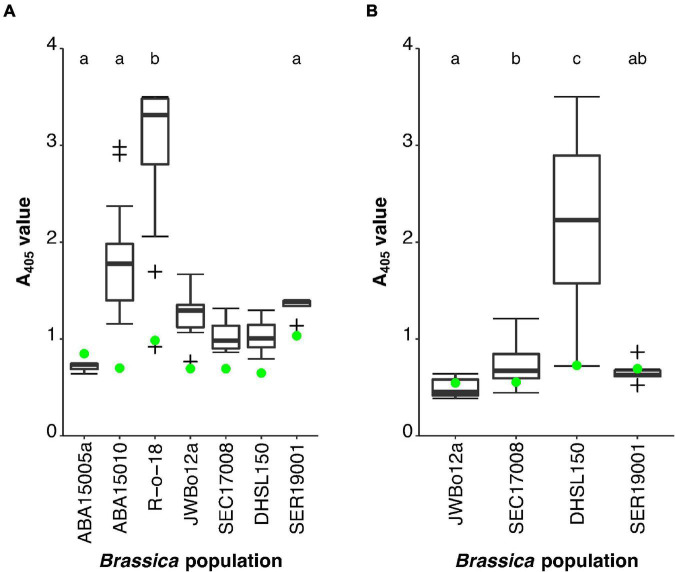
Turnip yellows virus (TuYV) phenotyping of resynthesised allotetraploid AACC S_1_ population SER19001 alongside TuYV-resistant and -susceptible *B. rapa* and *B. oleracea* lines. ABA15005a (*B. rapa*) (*n* = 3) and JWBo12a (*B. oleracea*) (*n* = 18) were the S_1_ populations TuYV-resistant parental lines of SER19001 (*n* = 5). R-o-18 (*B. rapa*) (*n* = 18) and DHSL150 (*B. oleracea*) (*n* = 12) were the TuYV-susceptible parental lines of the within-species mapping populations. ABA15010 (*B. rapa*) (*n* = 17) and SEC17008 (*B. oleracea*) (*n* = 18) were the F_1_ populations. Two plants from each line were not challenged with TuYV and were used as healthy controls. The mean healthy control value for each population is indicated (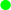). Populations with mean ranks not significantly different from one another are represented by the same lowercase letter (Dunn’s test with Bonferroni correction for multiple pairwise comparisons at significance level *p* = 0.05). **(A)** Enzyme-Linked Immunosorbent Assay (ELISA) A_405_ values 4 weeks post TuYV challenge. The *B. oleracea* lines were not included in the statistical analysis at this time point. **(B)** ELISA A_405_ values 11 weeks post TuYV challenge. Outliers are indicated by +.

At four weeks post-challenge, TuYV titre in the *B. oleracea* lines had not yet accumulated to detectable levels by TAS-ELISA (Figure 3A). This did not occur until 11 weeks post-challenge (Figure 3B). Therefore, the *B. oleracea* lines were omitted from the statistical analyses carried out 4 weeks post-challenge. Of the remaining four lines, the A_405_ values for R-o-18 (*p* < 0.001) and SER19001 (*p* = 0.020) did not fit normal distributions, despite transformations in, Shapiro–Wilk tests. Therefore, a Kruskal–Wallis test was carried out, which showed that there were significant differences in A_405_ values between the *Brassica* lines, *H*(3) = 27.769, *p* < 0.001 (Figure 3A). Subsequent Dunn’s pairwise comparisons with Bonferroni correction identified that the S_1_ population of the TuYV-resistant *B. rapa* parental line ABA15005a (mean rank = 2.00, *n* = 3) had a significantly lower mean A_405_ rank than the TuYV-susceptible *B. rapa* line R-o-18 (mean rank = 32.22, *n* = 18; *p* = 0.001). The resynthesised allotetraploid AACC S_1_ population SER19001 (mean rank = 6.80, *n* = 5) did not have a significantly different mean rank to ABA15005a (*p* = 1.000), but it had a significantly lower mean rank than R-o-18 (*p* < 0.001), indicating that TuYV resistance had been successfully integrated into the population from one/both of its parents. The *B. rapa* F_1_ population had an intermediate range of A_405_ values compared to its parental lines. Its mean rank (19.18, *n* = 17) was not significantly different from that of the resistant parental line ABA15005a (*p* = 0.173) but was significantly lower than the susceptible parental line R-o-18 (*p* = 0.013).

At 11 weeks post-challenge, the A_405_ values for JWBo12a (*p* < 0.001), the *B. oleracea* F_1_ population (*p* = 0.016), and SER19001 (*p* < 0.001) did not fit normal distributions despite transformations in Shapiro–Wilk tests. A Kruskal–Wallis test was carried out, which showed that there were significant differences in A_405_ values between the *Brassica* lines, *H*(3) = 31.856, *p* < 0.001 (Figure 3B). Subsequent Dunn’s pairwise comparisons with Bonferroni correction identified that the S_1_ population of the TuYV-resistant *B. oleracea* parental line JWBo12a (mean rank = 16.69, *n* = 18) had a significantly lower mean A_405_ rank than the TuYV-susceptible *B. oleracea* line DHSL150 (mean rank = 44.75, *n* = 12; *p* < 0.001). The resynthesised allotetraploid AACC S_1_ population SER19001 (mean rank = 11.00, *n* = 5) did not have a significantly different mean rank to JWBo12a (*p* = 1.000) but did have a significantly lower mean rank than DHSL150 (*p* < 0.001). This confirmed that TuYV resistance had been successfully integrated into the allotetraploid population from one/both parents. Like the *B. rapa* F_1_ population, the *B. oleracea* F_1_ population (mean rank = 29.92, *n* = 18) had an intermediate range of A_405_ values relative to the parental lines. It had a mean rank, which was significantly higher than that of the resistant parental line JWBo12a (*p* = 0.048) but was significantly lower than the susceptible parental line DHSL150 (*p* = 0.047).

#### Genotyping of Resynthesised Allotetraploid Plants for Turnip Yellows Virus Resistance

As phenotyping of the resynthesised allotetraploid AACC line SER19001 indicated that the population possessed TuYV resistance from one/both of the diploid parental lines ABA15005 and JWBo12, individuals from SER19001 were genotyped with informative SNP markers. Markers Bn-A02-7840077, Bn-A06-18369013, and Bn-scaff_16082_1-p278297 positioned on or closest to the peak LOD scores on chromosome A02, A06, and C05, respectively, strongly segregated with resistance in the mapping populations (Supplementary Table 1) and were used to determine if resistance alleles had been introgressed from both parents. The allotetraploid plants were homozygous for all these resistance alleles (Table 4).

**TABLE 4:** Genotype of S_1_ individuals SER19.1–7 from population SER19001 derived from the interspecific cross of TuYV-resistant lines ABA15005a (*B. rapa*, individual SE4.222) and JWBo12 (*B. oleracea*, individual DK1.134) and susceptible diploid *B. rapa* (R-o-18) and *B. oleracea* (DHSL150) plants at TuYV resistance-linked markers identified from QTL mapping.

Plant individual	Genome	TuYV resistance status	Genotype at TuYV-resistance linked marker (QTL position):		
			Bn-A02-p7840077 (chr. A02)	Bn-A06-p18369013 (chr. A06)	Bn-scaff_16082_1-p278297 (chr. C05)
SE4.222	AA	Resistant	A/A	T/T	–
R-o-18	AA	Susceptible	G/G	C/C	–
DK1.134	CC	Resistant	–	–	C/C
DHSL150	CC	Susceptible	–	–	A/A
SER19.1	AACC	Resistant	A/A	T/T	C/C
SER19.2	AACC	Resistant	A/A	T/T	C/C
SER19.3	AACC	Resistant	A/A	T/T	C/C
SER19.4	AACC	Resistant	A/A	T/T	C/C
SER19.5	AACC	Resistant	A/A	T/T	C/C
SER19.6	AACC	Resistant	A/A	T/T	C/C
SER19.7	AACC	Resistant	A/A	T/T	C/C

## Discussion

Aphid-borne TuYV is a widespread, yield-reducing pathogen of oilseed and vegetable brassicas. Due to the evolution of insecticide resistance in vectors and the neonicotinoid ban in the EU (The European Commission [EC], 2013), it has become necessary to exploit host resistance to control TuYV. At present, there are only two sources of genetic resistance to the virus in *B. napus*, and both have been mapped to co-locating QTLs on chromosome A04 (Dreyer et al., 2001; Juergens et al., 2010; Hackenberg et al., 2020). Here, we report the characterisation and genetic mapping of new TuYV resistance in *B. rapa* and *B. oleracea* and their successful introgression into a *B. napus* line by resynthesis. This is the first *B. napus* line to possess dual resistance to the virus in the A and C *Brassica* genomes and the first report of resistance mapped to the C genome.

### Characterisation and Mapping of Turnip Yellows Virus Resistance in *B. rapa* and *B. oleracea*

Turnip yellows virus resistance was identified in *B. rapa* ssp. *pekinensis* line ABA15005 and *B. oleracea* line JWBo12 and mapped in segregating backcross populations. Individuals in these populations showed a continuous spread of viral titres (Figures 1A, 2A), suggesting that both the ABA15005 and JWBo12 TuYV resistances were quantitative like previously described resistances to TuYV in brassicas (Dreyer et al., 2001; Hackenberg et al., 2020). Quantitative resistances tend to be more durable than complete resistances, as they do not exert strong pressure on pathogen evolution to virulence (Corwin and Kliebenstein, 2017). To date, no complete forms of TuYV resistance have been identified. The phenotypes and presence of resistant individuals within the segregating backcross populations indicate that both resistances are dominant and quantitative. The majority of resistances characterised to other members of the *Solemoviridae*, including to the type member potato leafroll virus (PLRV), cucurbit aphid-borne yellows virus (CABYV), and barley yellows dwarf virus (BYDV), have also been described as quantitative and dominantly inherited (Collins et al., 1996; Marczewski et al., 2001; Kassem et al., 2015).

F_1_ individuals derived from ABA15005 and JWBo12 showed a range of viral titres intermediate to the TuYV-resistant and -susceptible parental lines (Figure 3), suggesting that the ABA15005 and JWBo12 resistances are partially dominant/quantitative. Partial dominance can reduce the power of QTL analysis when using backcross populations as heterozygous individuals have intermediate phenotypes compared to homozygous-resistant and -susceptible individuals, whereas, with completely dominant resistance, heterozygotes have the same phenotype as the homozygous-resistant individuals. The *B. oleracea* resistance appeared to be more dominant than the *B. rapa* resistance. To counteract the effect of the weaker dominance on the analysis, a BC_2_ population, which had undergone more genomic recombination compared to a BC_1_ population, was used for QTL mapping in *B. rapa*. The *B. oleracea* line JWBo12 took nearly 3 years to flower, so it was only possible to produce a BC_1_ population for QTL mapping. However, JWBo12 mapping was less affected by partial dominance. It will be important for breeders to consider the effect of partial dominance when they introgress TuYV resistances from ABA15005 and JWBo12 into commercial OSR types, as they will be less effective in F_1_ hybrids where only one parent possesses the resistance.

For TuYV resistance originating from *B. rapa* line ABA15005, a significant full two-QTL model was detected by both one- and two-dimensional IM. This model involved positively interacting QTLs, one on chromosome A02 (37.0–53.0 cM) and the other on chromosome A06 (69.0–103.5 cM). The QTL on A02 and A06 explained 23.1% and 17.2% of the phenotypic variation, respectively (Figure 1B and Table 1). Combined, the QTLs explained 40.3% of the phenotypic variation, a percentage similar to those reported for the single-QTL TuYV resistances in “Yudal” (36.0%) and “R54” (50.4%) (Dreyer et al., 2001; Hackenberg et al., 2020). Based on the most recently annotated *B. rapa* Chiifu-401-42 reference genome (NCBI: GCA_000309985.3), the QTL interval on A02 is 6.9 Mbp in length and contains 1,052 genes, and the QTL interval on A06 is 4.0 Mbp in length and contains 795 genes. An additional two-QTL model was identified by two-dimensional IM, which involved the same QTL on A02 but a different additive and non-interacting QTL on A06 at 18.6 cM, which did not meet significance during one-dimension IM. This suggests that TuYV resistance in ABA15005 is controlled by two major QTLs on chromosomes A02 and A06, and that, potentially, a third smaller contributing QTL at the beginning of chromosome A06 might also contribute.

One-dimensional IM of TuYV resistance in *B. oleracea* line JWBo12 detected two QTLs with overlapping 1.5 LOD intervals on chromosome C05 (41.0–88.0 cM) (Figure 2B). Based on the annotated *B. oleracea* TO1000 reference genome (NCBI: GCA_000695525.1), the QTL interval on chromosome C05 is 37.0 Mbp in length and contains 3,773 genes. The first QTL peak in the interval positioned at 43.7 cM explained 11.5% of the phenotypic variation, and the second, positioned relatively closely at 81.4 cM, explained 10.6% (Table 1). However, no significant two-QTL models were detected by two-dimensional IM to support the findings of the one-dimensional IM. The double QTL peak observed could be an artefact of a reduced call rate of 98.9% for markers in the QTL interval compared to the average call rate of 99.5% for all markers in the analysis. This, in turn, could have led to reduced QTL detection power in the region. It could also be a result of the incorrect linkage assembly and/or structural variation, although there was no evidence of this when inspecting the pairwise comparisons of recombination fractions and LOD scores (Supplementary Figure 7), and the marker order in the linkage map corresponded to the physical order of markers in the *Brassica* C pangenome (He et al., 2015). The backcross population used for mapping TuYV resistance in JWBo12 showed a continuous distribution of viral titres and not the 1:1 segregation expected for a single completely dominant QTL (Figure 2A). This may be due to the quantitative nature and partial dominance of the resistance but may also have been influenced by environmental conditions at the time of the experiment and/or the influence of minor contributing QTLs that did not reach the significance threshold in the analyses. A similar, continuous range of viral titres was also seen in the backcross population used to map TuYV resistance in ABA15005 (Figure 1A), which was shown to be controlled by multiple QTLs. A QTL on chromosome C05 had been previously detected when mapping the “Yudal” resistance, but this QTL had only a minor influence on the trait (Hackenberg et al., 2020). Therefore, the resistance in JWBo12 is the first characterised and mapped major TuYV resistance QTL in the *Brassica* C genome. In addition to exploiting this resistance in OSR, it can also be exploited in vegetable brassicas, where the virus has been reported to reduce yields by as much as 65% in Brussels sprouts (Walsh et al., 2011).

### Introgression of Novel Turnip Yellows Virus Resistances Into *B. napus*

An allotetraploid AACC line was successfully resynthesised from the interspecific cross between the two TuYV-resistant lines ABA15005 (*B. rapa*) and JWBo12 (*B. oleracea*). The interspecific cross was more efficient when the *B. rapa* line was the female parent (7.4 ovules/siliqua) compared to when it was the male parent (0.7 ovules/siliqua). It has been suggested that the most efficient interspecific crosses are those where the female parent of the cross is the one with the greatest number of chromosomes (Mohammad and Sikka, 1940; Lu et al., 2001; Malek et al., 2012), which was the case here. Previously, much higher but broad-ranging *B. napus* resynthesis efficiencies have been reported than those in this study (Lu et al., 2001; Zhang et al., 2004; Malek et al., 2012). This is likely a result of the compatibility of the specific parental genotypes used in the interspecific crosses. It could also be a result of differences in the tissue culture methods used. In total, three plantlets (RP1–RP3) were regenerated from the interspecific cross between ABA15005 and JWBo12, and from these, 12 cuttings (C1–C12) were taken and treated with colchicine. Ploidy testing of the regenerated plantlets and cuttings identified that they were true AC hybrids and mixoploid (1–16 × AC) (Table 2); to stabilise ploidy, plants were self-pollinated. Only one cutting produced fertile flowers, which were subsequently self-pollinated to produce the resynthesised allotetraploid AACC S_1_ population SER19001. Individuals from this population had the allotetraploid genome AACC (Table 3). Other cuttings were sterile, probably due to failure in chromosome multiplication; hybrids possessing a diploid AC genome have been reported to be 99–100% sterile (Malek et al., 2012).

When phenotyped at four and 11 weeks post-challenge with viruliferous aphids, the resynthesised allotetraploid AACC S_1_ population SER19001 was shown to be uniformly resistant to TuYV. There were no significant differences in the strength of resistances between SER19001 and S_1_ populations of its diploid-resistant parental lines ABA15005a and JWBo12a, and it had significantly lower viral titres than the susceptible *B. rapa* and *B. oleracea* control lines (Figure 3). Subsequent genotyping of SER19001 with TuYV resistance-linked markers identified from QTL mapping in the progenitor species suggested that resistance had been successfully introgressed from both parental lines, making SER19001 the first *B. napus* population to possess dual resistance to TuYV in the A and C genomes. Although the phenotyping results suggest that possessing dual resistance may be no stronger, in terms of reducing viral titre, than the single resistances in ABA15005 and JWBo12, it is likely to be more durable; having dual or “stacked” resistance is likely to reduce the evolutionary pressure for resistance-breaking isolates of TuYV compared to a single resistance.

### Conclusion and Future Study

In conclusion, novel TuYV resistances have successfully been mapped in *B. rapa* and *B. oleracea* and introgressed into the an allotetraploid AACC line by resynthesis. This will help to broaden and improve the very limited TuYV resistance base in OSR. The resynthesised allotetraploid AACC line is the first to possess dual resistance to the virus from both the A and C genomes. Going forward, fine mapping in the progenitor species will help identify markers that are more closely linked to TuYV resistance. In turn, these markers can be used to introgress the resistances into commercial OSR and vegetable brassica types by marker-assisted selection. Fine mapping will also help narrow down the list of candidate TuYV resistance genes. As, currently, no resistance genes have been identified to TuYV or any other members of the *Solemoviridae* family. Whilst the new resistances provide protection against an isolate of TuYV belonging to the most abundant phylogenetic group, it is still to be seen whether they will provide protection against other strains of the virus and how they compare in strength and durability to existing commercial resistances, individually and combined.

## Data Availability Statement

The original contributions presented in the study are included in the article/Supplementary Material, further inquiries can be directed to the corresponding authors.

## Author Contributions

JAW, GCB, VG, GM, and SFG conceived the *B. rapa* and *B. oleracea* TuYV resistance mapping and *B. napus* resynthesis experiments. SFG conducted the experiments, constructed genetic linkage maps and carried out QTL analysis on the BC populations, supported by GRT and DH, carried out *B. napus* resynthesis and the subsequent phenotyping of the resynthesised plants for TuYV resistance, and genotyped resynthesised allotetraploid AACC plants with TuYV resistance-linked markers identified from QTL mapping in the progenitor species. JB and DE genotyped the *B. rapa* BC_2_ population and *B. oleracea* BC_1_ population. SFG and JAW wrote the manuscript. All authors read and reviewed the manuscript.

## Conflict of Interest

VG and GM were employed by the company Limagrain UK Ltd. The remaining authors declare that this study received funding from Limagrain UK Ltd. The funder had the following involvement in the study: they agreed to the experimentation, provided feedback on progress and were involved in the conception of other aspects of the Ph.D. studies.

## Publisher’s Note

All claims expressed in this article are solely those of the authors and do not necessarily represent those of their affiliated organizations, or those of the publisher, the editors and the reviewers. Any product that may be evaluated in this article, or claim that may be made by its manufacturer, is not guaranteed or endorsed by the publisher.
