# Supplementary material for: Quantitative Trait Locus Mapping of Resistance to Turnip Yellows Virus in Brassica rapa and Brassica oleracea and Introgression of These Resistances by Resynthesis Into Allotetraploid Plants for Deployment in Brassica napus
Source: Front Plant Sci. 2021 Dec 10;12:781385. doi: 10.3389/fpls.2021.781385 (PMC8703028; doi:10.3389/fpls.2021.781385)
Supplement: Supplementary file 1 [file Data_Sheet_1.docx]

Supplementary Material

# Supplementary Figures

R-o-18♀ (TuYV-susceptible)

ABA15005 ♂ (TuYV-resistant)

x

R-o-18♀

ABA15010 ♂ (TuYV-resistant F_1_)

x

ABA15010 ♂ (TuYV-resistant BC_1_)

x

R-o-18♀

SEA17016 (BC_2_, QTL mapping population)

ABA15005a

 ⨂

**Supplementary Figure 1. Crossing strategy for the production of *Brassica rapa* BC_2_ mapping population SEA17016.**

DHSL150♀ (TuYV-susceptible)

JWBo12a

JWBo12 ♂ (TuYV-resistant)

x

SEC17008 ♂ (TuYV-resistant F_1_)

DHSL150♀

SEC18031♀ (BC_1_, QTL mapping population)

 ⨂

x

**Supplementary Figure 2. Crossing strategy for the production of *Brassica oleracea* BC_1_ mapping population SEC18031.**

**
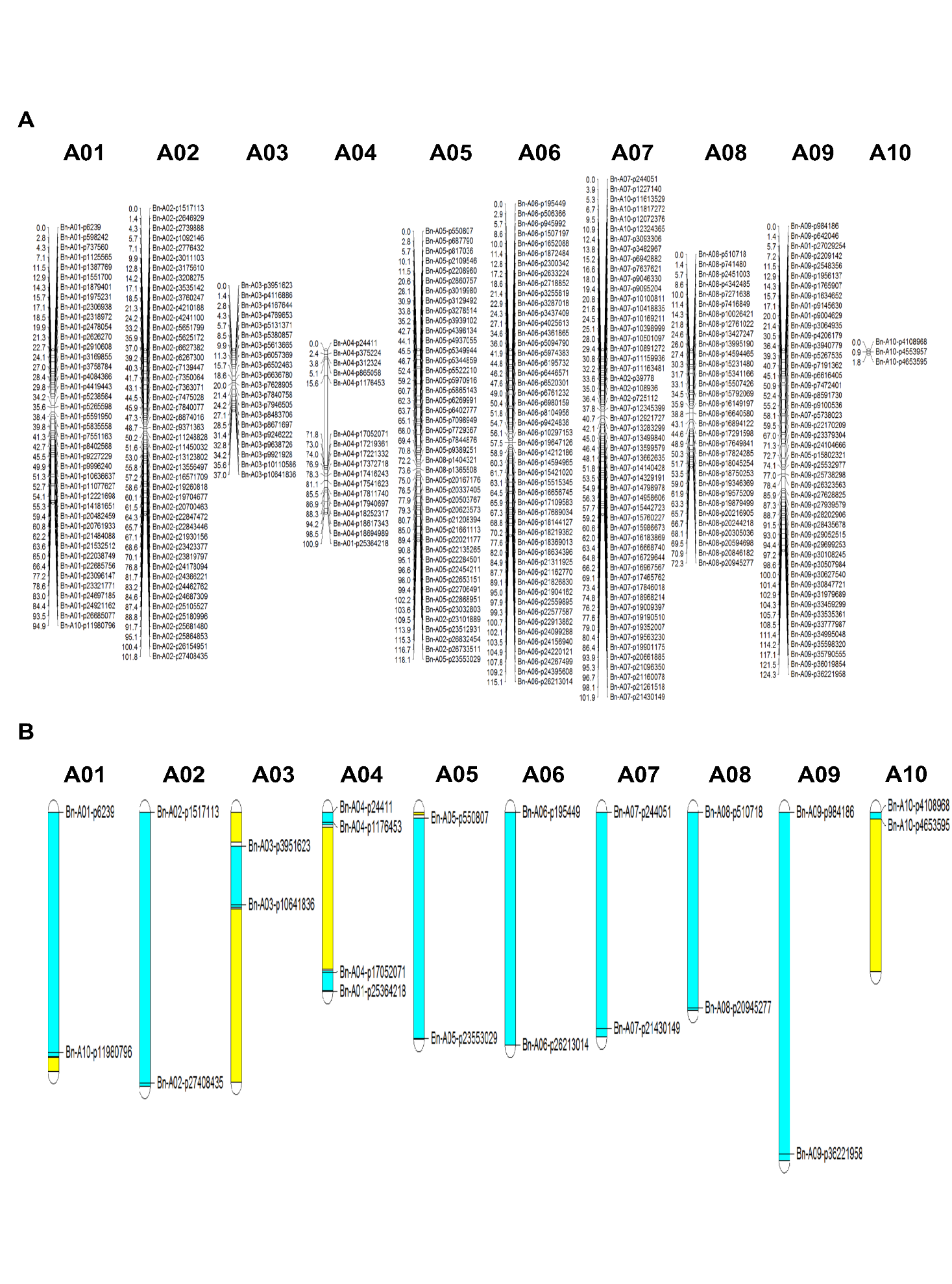
Supplementary Figure 3. Minimal genetic linkage map of *Brassica rapa* BC_2_ population SEA17016. A.** Genetic linkage map of SEA17016. Markers in this map only segregate in regions where the BC_1_ plant had a heterozygous genotype. **B.** Genotype of the BC_1_ individual used to produce SEA17016 across the physical map of polymorphic markers identified between the parental lines R-o-18 and ABA15005. SEA17016 was produced from the cross R-o-18 x BC_1_. Regions of the map where the BC_1_ plant was homozygous for the R-o-18 allele are in yellow and regions where it was heterozygous are in blue.

**

**

**Supplementary Figure 4. Comparison of non-parametric and parametric quantitative trail locus (QTL) analyses of turnip yellows virus resistance in *Brassica rapa* BC_2_ population SEA17016.** Non-parametric, Kruskal-Wallis one-dimensional interval mapping was carried out on untransformed A_405_ values (black) and parametric, Haley-Knott one-dimensional interval mapping was carried out on transformed log_10_(A_405_) values (grey). The genome-wide significance LOD thresholds ($\alpha\leq$0.05) of 1000 permutations are indicated by a dashed horizontal black line for the non-parametric analysis and a dashed horizontal grey line for the parametric analysis.

**
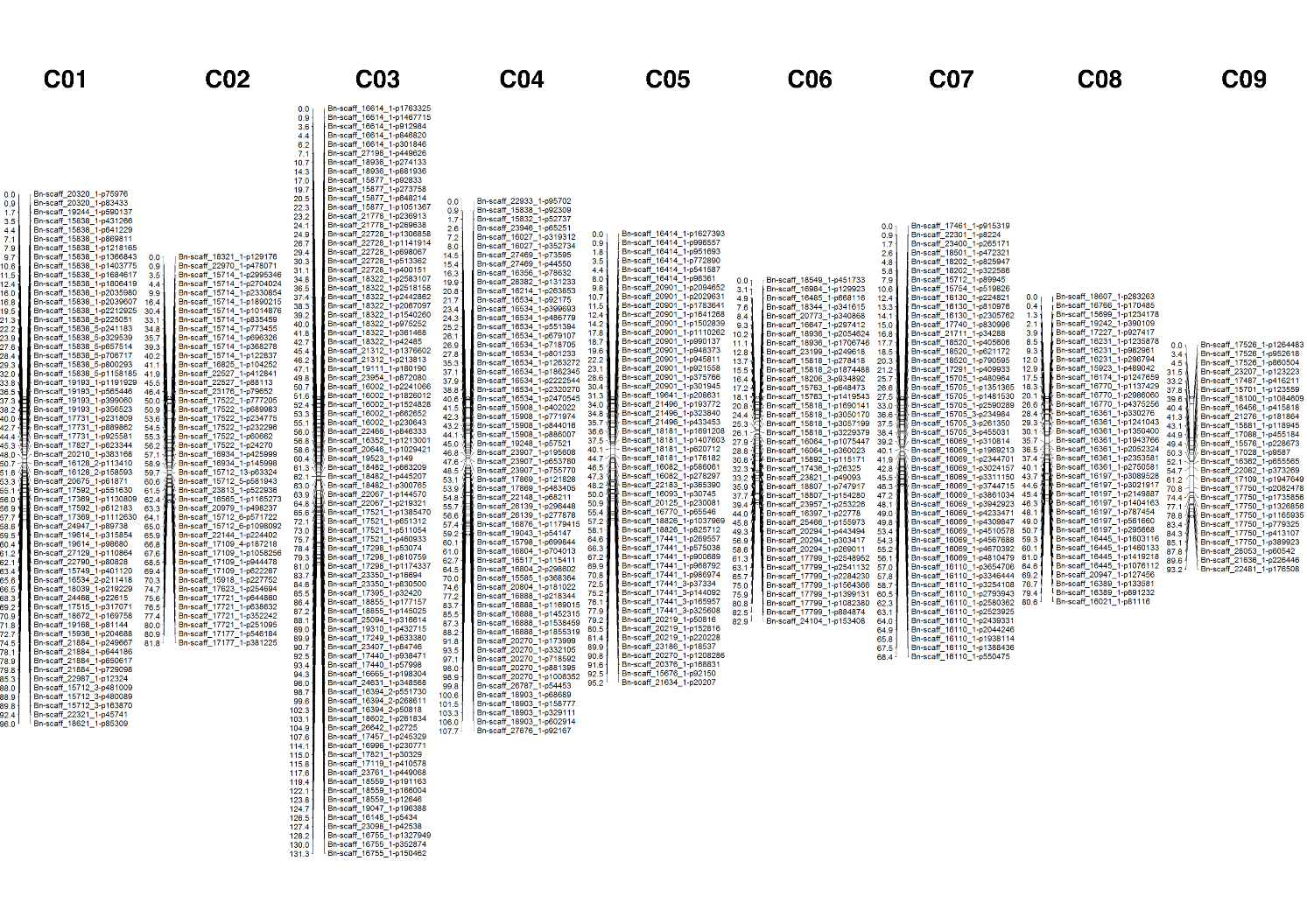
Supplementary Figure 5. Minimal genetic linkage map of *Brassica oleracea* BC_1_ population SEC18031.**





**Supplementary Figure 6. Comparison of non-parametric and parametric quantitative trail locus (QTL) analyses of turnip yellows virus resistance in *Brassica oleracea* BC_1_ population SEC18031.** Non-parametric, Kruskal-Wallis one-dimensional interval mapping was carried out on untransformed A_405_ values (black). Parametric, Haley-Knott one-dimensional interval mapping was carried out on transformed log_10_(A_405_) values (grey). The genome-wide significance LOD thresholds ($\alpha\leq$0.05) of 1000 permutations are indicated by a dashed horizontal black line for the non-parametric analysis and a dashed horizontal grey line for the parametric analysis.

**Supplementary Table 1. Genotype of individuals with the ten lowest A_405_ values [turnip yellows virus (TuYV)-resistant) and highest A_405_ values (TuYV-susceptible) from the *Brassica rapa* and *Brassica oleracea* mapping populations SEA17016 and SEC18031, respectively at TuYV resistance-linked markers. SEA17016 was derived from the parental cross of R-o-18 (TuYV-susceptible) x ABA15005 (TuYV-resistant) and SEC18031 was derived from the parental cross DHSL150 (TuYV-susceptible) x JWBo12 (TuYV-resistant). Possession of the resistance allele is highlighted in blue.**

| **Population** | **Plant individual** | **A_405_ value** | **TuYV resistance status** | **Genotype at TuYV-resistance linked marker (position):** | | |
| --- | --- | --- | --- | --- | --- | --- |
|  |  |  |  | **Bn-A02-p7840077 (chr. A02)** | **Bn-A06-p18369013 (chr. A06)** | **Bn-scaff_16082_1-p278297 (chr. C05)** |
| ABA15005 | SE4.222 | - | Resistant | A/A | T/T | - |
| R-o-18 | R-o-18 | - | Susceptible | G/G | C/C | - |
| *B. rapa* | 15.16 | 0.185 | Resistant | G/G | C/T | - |
| SEA17016 | 15.38 | 0.197 | Resistant | - | C/T | - |
|  | 15.121 | 0.207 | Resistant | A/G | C/T | - |
|  | 15.124 | 0.219 | Resistant | A/G | C/T | - |
|  | 15.126 | 0.233 | Resistant | A/G | C/T | - |
|  | 15.138 | 0.243 | Resistant | A/G | C/T | - |
|  | 15.105 | 0.252 | Resistant | A/G | C/T | - |
|  | 15.100 | 0.255 | Resistant | A/G | C/T | - |
|  | 15.70 | 0.273 | Resistant | A/G | C/T | - |
|  | 15.113 | 0.279 | Resistant | A/G | C/T | - |
|  | 15.156 | 2.977 | Susceptible | A/G | C/C | - |
|  | 15.56 | 2.826 | Susceptible | G/G | C/C | - |
|  | 15.196 | 2.632 | Susceptible | G/G | C/C | - |
|  | 15.189 | 2.583 | Susceptible | G/G | C/C | - |
|  | 15.50 | 2.228 | Susceptible | G/G | C/C | - |
|  | 15.4 | 2.135 | Susceptible | G/G | C/T | - |
|  | 15.69 | 1.766 | Susceptible | G/G | C/C | - |
|  | 15.135 | 1.739 | Susceptible | G/G | C/C | - |
|  | 15.53 | 1.656 | Susceptible | G/G | C/C | - |
|  | 15.49 | 1.611 | Susceptible | G/G | C/C | - |
| JWBo12 | DK1.134 | - | Resistant | - | - | C/C |
| DHSL150 | DHSL150 | - | Susceptible | - | - | A/A |
| *B. oleracea* | 16.171 | 0.566 | Resistant | - | - | A/C |
| SEC18031 | 16.60 | 0.638 | Resistant | - | - | A/C |
|  | 16.82 | 0.648 | Resistant | - | - | A/C |
|  | 16.70 | 0.658 | Resistant | - | - | A/A |
|  | 16.111 | 0.669 | Resistant | - | - | A/A |
|  | 16.100 | 0.702 | Resistant | - | - | A/A |
|  | 16.68 | 0.740 | Resistant | - | - | A/C |
|  | 16.67 | 0.754 | Resistant | - | - | A/A |
|  | 16.89 | 0.762 | Resistant | - | - | A/C |
|  | 16.189 | 0.771 | Resistant | - | - | A/C |
|  | 16.176 | 3.500 | Susceptible | - | - | A/C |
|  | 16.187 | 3.121 | Susceptible | - | - | A/A |
|  | 16.126 | 2.700 | Susceptible | - | - | A/A |
|  | 16.162 | 2.603 | Susceptible | - | - | A/C |
|  | 16.85 | 2.581 | Susceptible | - | - | A/A |
|  | 16.5 | 2.379 | Susceptible | - | - | A/A |
|  | 16.188 | 2.190 | Susceptible | - | - | A/A |
|  | 16.73 | 2.131 | Susceptible | - | - | A/A |
|  | 16.174 | 2.047 | Susceptible | - | - | A/A |
|  | 16.177 | 2.035 | Susceptible | - | - | A/A |

**
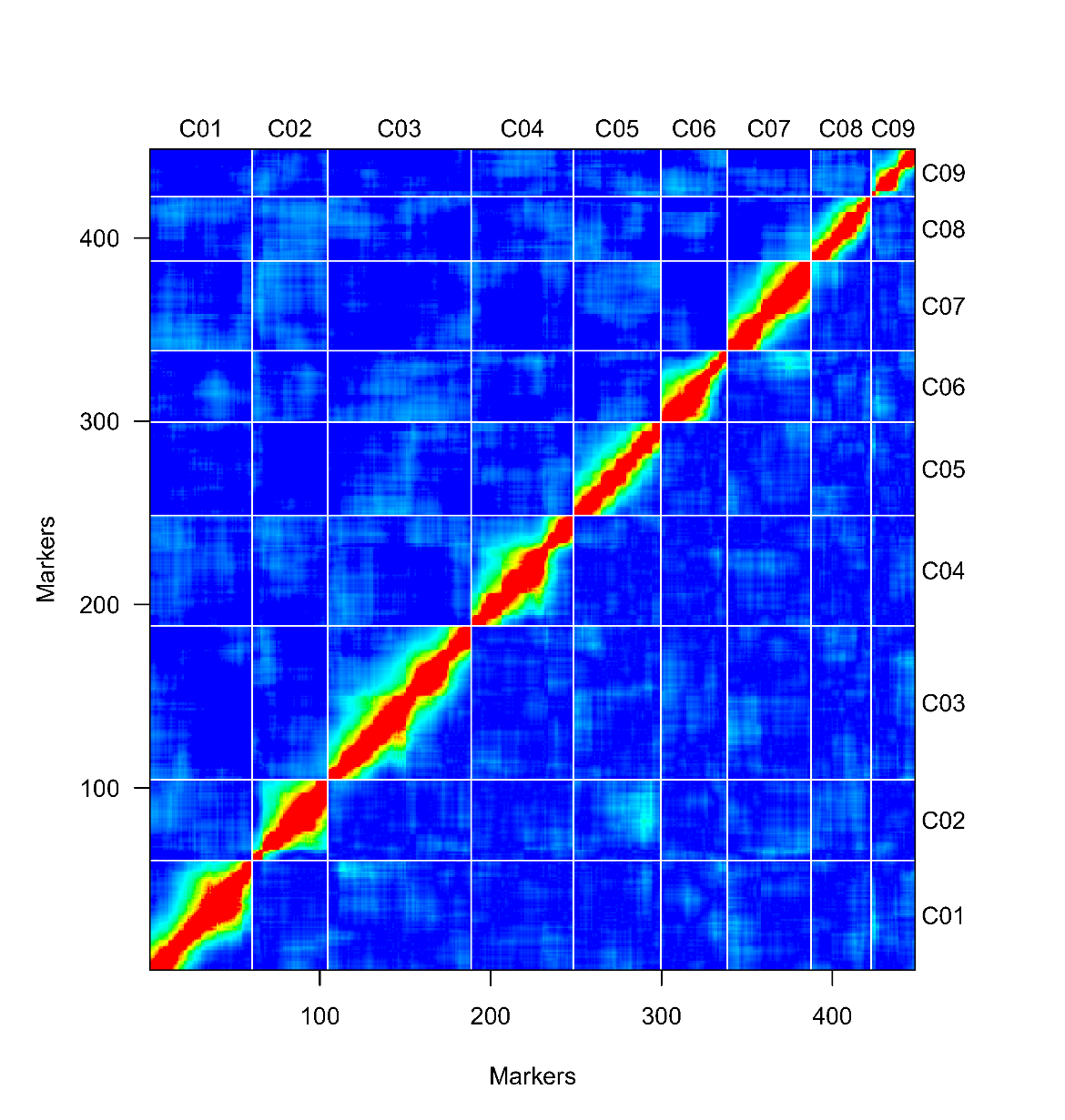
**
**Supplementary Figure 7. Pairwise comparisons of recombination fractions (top left triangle) and LOD scores (bottom right triangle) for each marker in the minimal genetic map constructed for *Brassica oleracea* BC_1_ population SEC18031.**

**
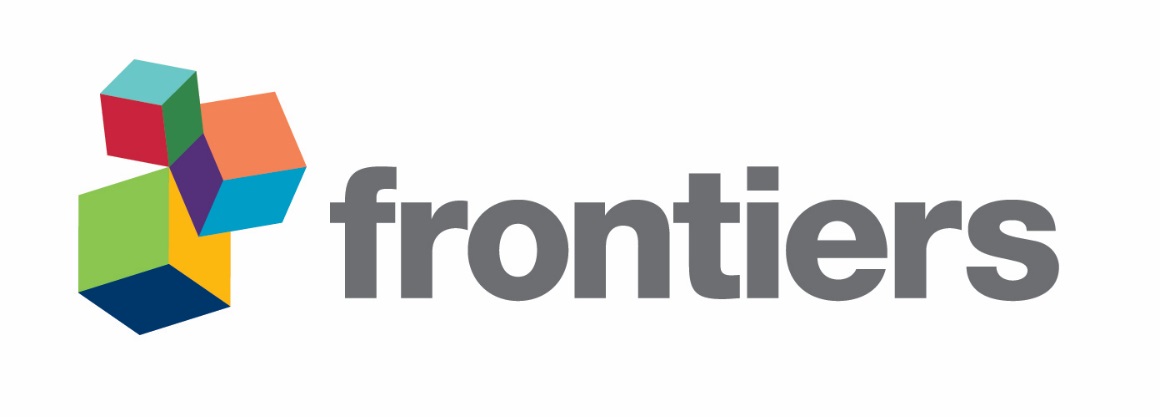
**
